# ASCOT: a text mining-based web-service for efficient search and assisted creation of clinical trials

**DOI:** 10.1186/1472-6947-12-S1-S3

**Published:** 2012-04-30

**Authors:** Ioannis Korkontzelos, Tingting Mu, Sophia Ananiadou

**Affiliations:** 1 National Centre for Text Mining & School of Computer Science, The University of Manchester, Manchester, M1 7DN, UK

## Abstract

Clinical trials are mandatory protocols describing medical research on humans and among the most valuable sources of medical practice evidence. Searching for trials relevant to some query is laborious due to the immense number of existing protocols. Apart from search, writing new trials includes composing detailed eligibility criteria, which might be time-consuming, especially for new researchers. In this paper we present *ASCOT*, an efficient search application customised for clinical trials. *ASCOT *uses text mining and data mining methods to enrich clinical trials with metadata, that in turn serve as effective tools to narrow down search. In addition, *ASCOT *integrates a component for recommending eligibility criteria based on a set of selected protocols.

## Introduction

Clinical trials are health-related research studies on humans. A description of a clinical trial follows a pre-defined protocol and contains various information about the study: its title, the condition under inspection, the target, the characteristics of patients that can participate, details about the institutions that accomplished the study, etc. Clinical trials are among the most valuable sources for guiding evidence-based medical practice and designing new trials. However, the vast and rapidly growing number of existing trails hinders the effective use of this information. Significant effort has been devoted to efficient search applications for clinical trials, mainly by various registries, e.g. the *UK Clinical Trials Gateway *http://www.ukctg.nihr.ac.uk, *Free International Standard Randomised Controlled Trials* (http://isrctn.org) and http://controlled-trials.com. However, none has attempted to enrich the free text descriptions with structured metadata able to provide search with extra discriminative powers needed for customised search.

One of the most difficult parts of creating a new clinical trial is listing the eligibility criteria, i.e. the characteristics of participants. At the same time, eligibility criteria can directly affect the quality of the experiment and its outcome. Although for experienced researchers composing the eligibility section of a trial might be straightforward, for inexperienced ones it would be time-consuming. It would imply searching for relevant trials and literature and then processing them.

In this paper we present *ASCOT (Assisting Search and Creation Of clinical Trials)*, an efficient search application customised to clinical trials that aims to address the information overload problem and to assist the creation of new protocols. *ASCOT *employs state-of-the-art text mining technologies, clustering and term extraction algorithms applied on large clinical trial collections. It is available at: http://www.nactem.ac.uk/clinical_trials.

In more detail, search begins with a textual query. Then, it can be narrowed down in a multitude of ways:

- by selecting values for properties that correspond to XML fields of the clinical trial protocols.

- by selecting one of the automatically induced and labelled clusters of clinical trial protocols.

- by selecting a *UMLS *or *SNOMED CT *concept to occur in the (inclusion or exclusion) eligibility criteria of the clinical trial protocols.

*The Unified Medical Language System (UMLS) *is a comprehensive thesaurus and ontology, grouping together many controlled biomedical vocabularies. It supports mapping among these vocabularies and several facilities for natural language processing. Examples of incorporated vocabularies are *ICD-10*, *MeSH*, *SNOMED CT *and the *Gene Ontology*.

*SNOMED CT *stands for "*S*ystematized *N*omenclature of *Me*dicine *C*linical *T*erms" and is a clinical terminology with comprehensive scientifically validated content. *SNOMED CT *is in use in more than fifty countries and is managed and maintained internationally by the International Health Terminology Standards Development Organisation (IHTSDO) and in the UK by the UK Terminology Centre (UKTC).

- by selecting one of the multiword terms, automatically extracted by the *C-Value *algorithm.

The above alternatives can be applied iteratively until the result seems satisfactory to the user. The user can select documents and add them to a separate selection board for further processing. Probable eligibility criteria based on the selected documents are generated automatically.

The paper is structured as follows: in section "Functionality", we discuss the user-side functionality of the application accompanied with explanatory screenshots. Section "System architecture" presents the architecture of offline and online processing components and their internal structure. In section "Related work", we discuss other approaches to clinical trials search and processing. Section "Conclusion" concludes the paper and summarises a few directions for future work.

### Functionality

The user interface is realised using *PASC *(*P*latform for Associative *S*earch and *C*lustering). *PASC *is a Java-based fully customisable search engine for developing semantic search applications, developed at the UK's National Centre for Text Mining (NaCTeM). It uses *Google Web Toolkit (GWT) *for the generation of the user interface and it can cooperate with any standalone search server, such as *Apache Lucene (lucene.apache.org)*, *Solr (lucene.apache.org/solr) *or any relational database that supports full-text indexing of text fields. *PASC *provides full-text search, complemented with a range of auxiliary search tools that can help make sense of large search result sets. Faceted search [1] allows the user to break down any search result dynamically into one of several topic hierarchies that can be explored independently. Topic clustering of the top ranked search results is supported and can be configured to use alternative clustering algorithms. *PASC *can cooperate with any other component that processes the whole or parts of the results further, e.g. identification of ontology concepts. Except for its fundamental search functionality, PASC provides mechanisms for the user to annotate their search results for their own purposes. *PASC *can be tightly coupled with *U-Compare *[2] to provide a complete solution combining analytic workflows and annotation search. *U-Compare *is a UIMA-based platform for building Natural Language Processing (NLP) and Text Mining (TM) workflows and provides access to the largest repository of interoperable text mining components. *U-Compare *was developed by *NaCTeM *and the *University of Tokyo *and is freely available at http://nactem.ac.uk/ucompare.

*PASC *has already been used for a variety of search projects such as *UKPMC* (http://ukpmc.ac.uk) and *ASSERT *http://www.nactem.ac.uk/assert. The latter aims to support the production of systematic reviews [3], a task very similar to filling in new clinical trial protocols.

**Figure 1:**
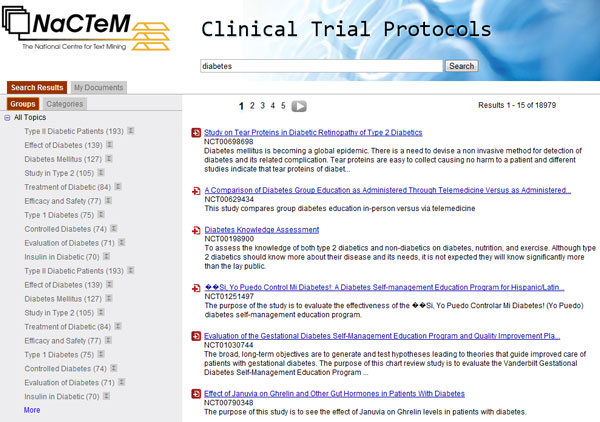
**Screenshot of the search interface**. Search results and view of "Groups".

Figure 1 shows a screenshot of the search environment. The query word "diabetes" has been submitted and 18,979 protocols were found to contain this word in their textual content. The title of each protocol, its unique identification number (e.g. *NCT00698698*) and a snippet from it that contains the query word is shown in the large right pane.

**Figure 2:**
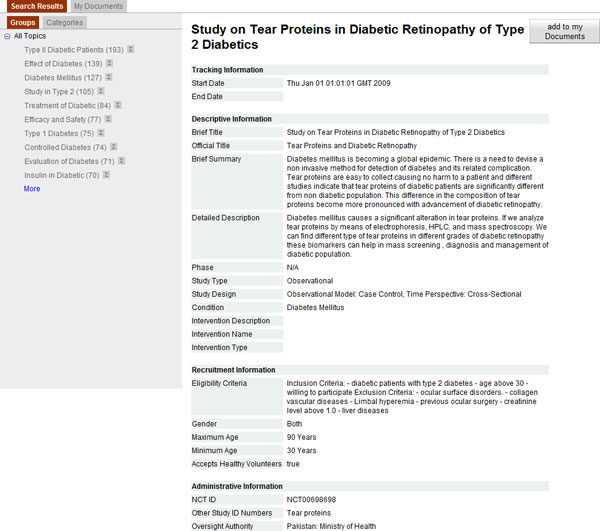
**Detailed view of a clinical trial protocol using ASCOT**.

Clicking on the title, reveals the content of the corresponding protocol. For example, Figure 2 illustrates the content of protocol *NCT00698698*. Selected XML fields are grouped into four categories: tracking, descriptive, recruitment and administrative information. Clicking the button *"add to my documents" *adds the corresponding document to a separate selection board for further processing. The same action can be performed via the small button (i.e. a combination of the symbol of a document and plus, in red colour) which is on the left of each protocol title in the search environment (Figure 1). In both of these manners, protocols can be selected in any step of many consecutive search operations.

**Figure 3:**
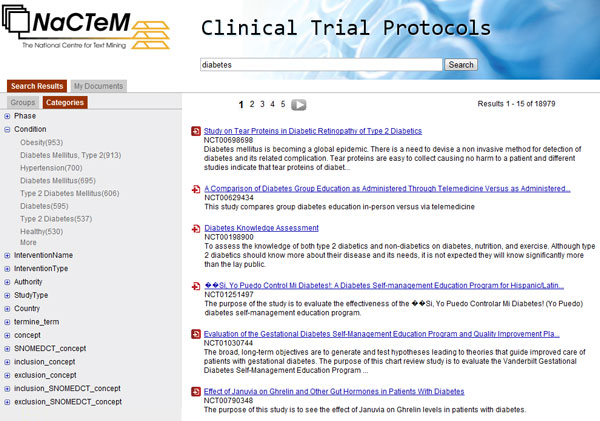
**Screenshot of the search interface**. Search results and view of "Categories".

The left hand side pane holds various features related to and describing the search results, useful to narrow down search. They are grouped in two sets which appear as window tabs: "groups" and "categories". Groups are labelled clusters that contain the protocols, i.e. the results of the current search, and are shown in Figure 1. Clusters are induced by the invoked clustering algorithm, discussed in subsection "Clusters and cluster labels". Categories, shown in Figure 3, are values of XML fields, automatically recognised terms and ontology concepts occurring in the eligibility criteria section. In detail, the categories are:

- ***Phase***: The trials at different phases have different purposes and exploit different questions. Example values: 0-4, 1/2, 2/3, N/A.

- ***Condition***: The conditions that clinical trials are conducted to treat, prevent, explore or detect. Examples: *HIV infections*, *breast cancer*, *obesity*, *leukemia*.

- ***Intervention Name***: The names of the interventions that are applied to the subjects of the trials. This XML field has values for interventional studies but not for observational studies. Example values: *placebo*, *laboratory biomarker analysis*, *radiation therapy*.

- ***Intervention Type***: The types of the above interventions. Examples: *drug*, *procedure*, *behavioral*, *radiation*.

- ***Authority***: The investigating authorities. Some examples are: *United States: Food and Drug Administration*, *Health Canada*, *Taiwan: Department of Health*.

- ***Study*_*Type***: Example values of this XML field: *interventional*, *observational*, *expanded access*, *N/A*.

- ***Country***: The countries where the procedures of a clinical trial take place. Although currently proocols from ClinicalTrials.gov are only included, *authority *and *country *are still informative. Within the U.S. the running authorities vary. Moreover, sometimes authorities of other countries participate.

- ***termine_term***: Multiword terms that occur in the textual contents of protocols, i.e. *brief summary*, *detailed description *and *eligibility criteria*, and have been identified using the *C-Value *algorithm, discussed in subsection "TERMINE: term extraction". Examples: *informed consent*, *upper limit*, *myocardial infraction*, *body mass*, *birth control*.

- ***concept***: UMLS concepts occurring in the eligibility criteria section. Examples: *age*, *patients*, *diagnosis*, *malignant neoplasms*, *pregnancy*, *male genre*.

- ***SNOMEDCT_concept***: *SNOMED CT *concepts occurring in the eligibility criteria section. These are a subset of *UMLS *concepts, above.

- ***inclusion_concept***: *UMLS *concepts occurring in the inclusion criteria. The inclusion and exclusion part of the eligibility criteria textual section are identified using a UIMA annotator (subsection "Eligibility criteria").

- ***exclusion_concept***: *UMLS *concepts occurring in the exclusion criteria.

- ***inclusion_SNOMEDCT_concept***: *SNOMED CT *concepts occurring in the inclusion criteria.

- ***exclusion_SNOMEDCT_concept***: *SNOMED CT *concepts occurring in the exclusion criteria.

**Figure 4:**
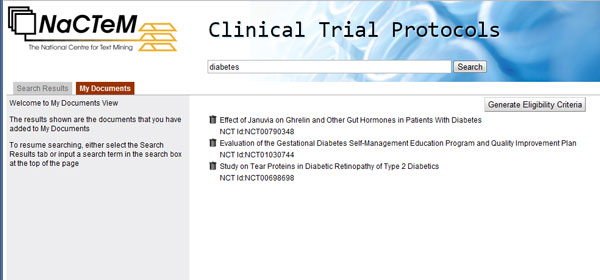
**View of "My documents"**. "My documents" is a separate panel for sorting documents selected by the user for further processing.

Figure 4 depicts a screenshot of the separate selection board called "My documents". It contains three protocols, which have been previously selected in Figure 1. This functionality is useful for saving the search results that fit users' needs and for further processing.

**Figure 5:**
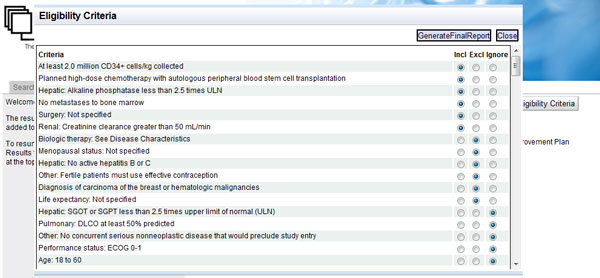
**The results of the eligibility criteria recommendation system**. The eligibility criteria recommendation system presents a list of criteria sorted in order of importance, as computed internally from the respective component based on the importance of the UMLS concepts that occur in each criterion. The user is able to forward desired criteria to the final report as inclusion or exclusion criteria, and ignore the remaining ones.

The button "Generate Eligibility Criteria" triggers the eligibility criteria recommendation system (sub-section "Recommendation system"). Figure 5 illustrates its output: a long list of recommended inclusion and exclusion eligibility criteria. Each recommended criterion can be selected as inclusion or exclusion criterion or can be ignored, via the radio button on its right. "Generate final report" concatenates the chosen inclusion and exclusion criteria forming text that can be copied to any editor.

### System architecture

**Figure 6:**
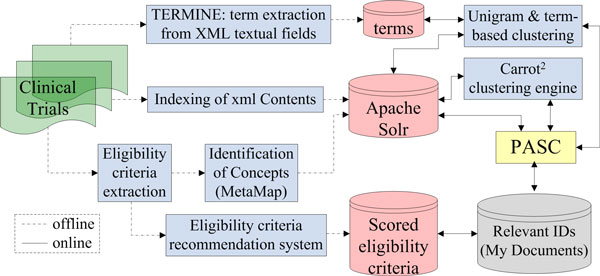
**A block diagram of ASCOT's architecture**. The green box represents the input documents, the yellow box denotes the search interface, blue boxes stand for processing components, red cylinders are repositories and the grey cylinder denotes the "My documents" panel, shown in Figure 4.

In the previous section, the user interface was described. In this section, we present the architecture of the system and discuss all invoked components. Figure 6 shows an overview.

The system consists of an online and an offline part. Offline processes do not depend on the search queries and thus can be executed before live search. Most of the offline components are resource and time intense. They are shown on the left of Figure 6 and are connected with dotted lines. In contrast, online components, which are shown on the right of Figure 6 and are connected with solid lines, serve operations that depend on each submitted search query or operation. The online processes are critical for the overall performance of the system and for this reason low complexity is a crucial design requirement. As discussed earlier, *PASC *is the central component of the online operation of the system. It contains the user interface, triggers other online components and queries the repositories of preprocessed data. These repositories, illustrated as red cylinders in Figure 6, hold the processing outcomes of the offline processes.

### Repository of protocols

The repository of clinical trial protocols, shown in green in Figure 6, is realised as a database table. Its entries represent protocols that are downloaded weekly from clinical trial registers in *Extensible Markup Language (XML) *format.

Currently, only ClinicalTrials.gov is used. It is a registry and results database of federally and privately supported clinical trials conducted in the United States and around the world. Other registries such as the *UK Clinical Trials Gateway*, *Free International Standard Randomised Controlled Trials *and controlled-trials.com are in the process of inclusion. They index information from a variety of publicly available national registers.

### TERMINE: term extraction

*Term extraction *is the identification of linguistic expressions denoting domain-specific concepts. Terms reflect the semantic content of the document in which they occur. For this reason, the contribution of terms to *ASCOT *is two-fold:

- directly, as a means of narrowing down search: as discussed in section "Functionality", the terms that occur in the search result documents consist one of the categories of Figure 3. They are sorted in decreasing order of frequency of occurrence. Clicking on a term from the list narrows the selection to documents that contain the chosen term.

- indirectly, as a clustering feature: *UTC*, one of the employed clustering and cluster labelling algorithms, uses unigrams and terms as features to represent documents.

Unsupervised term recognition typically uses linguistic filtering to identify term candidates [4] and then scores them according to some statistical measure. Measures that consider *nestedness *information, namely the frequencies of candidate terms occurring as subsequences of longer candidate terms (e.g *C-Value *[5] and the statistical barrier method [6]), have been shown to outperform measures that quantify the attachment strength among the constituents of a candidate term (e.g. frequency of co-occurrence and hypothesis testing statistics). Moreover, it has been experimentally shown that *C-Value *[5] is among the best performing measures [7]. For this reason, we choose to employ the *C-Value *measure in our pipeline. It exploits nestedness and scores candidate multiword terms considering:

- the total frequency of occurrence of the candidate term;

- the frequency of the candidate term as part of longer candidate terms;

- the number of these **distinct **longer candidates;

- the length of the candidate term (in tokens).

Nestedness is computed according to the following formula:

(1) $$N\left(\alpha \right)=\left\{\begin{array}{c} f\left(\alpha \right),\text{if }\alpha \text{ is not nested}\\ f\left(\alpha \right)-\frac{1}{|T_{\alpha }|}\underset{b\in T_{\alpha }}{\sum }f\left(b\right),\;\text{otherwise} \end{array}\right)$$

where α is the candidate term, *f *(α) is its frequency, *T_α _*is the set of candidate terms that contain α and | *T_α_*| is the cardinality of *T_α_*. In simple terms, formula 1 encodes the following intuitions:

- the more frequently a candidate term appears as a substring of other candidates, the less likely it is a valid term.

- the greater the number of **distinct **term candidates in which the target term candidate occurs as nested, the more likely it is a valid term.

The final *C-Value *score considers the length (|α|) of each candidate term (α) as well:

(2) $$C\text{ - }value\left(\alpha \right)={\text{log}}_{2}|\alpha |\times N\left(\alpha \right)$$

The *C-Value *method identifies term candidates based on part of speech (PoS) patterns. In our approach, we used the PoS assigned by the *GENIA tagger *http://www-tsujii.is.s.u-tokyo.ac.jp/GENIA/tagger, which is reported to achieve state-of-the-art performance both on newswire and biomedical corpora [8]. Identification of candidate terms is followed by the computation of *C-Value*, in length order, longest first. Candidates that satisfy a C-Value threshold (>1) are sorted in decreasing *C-Value *order.

### Eligibility criteria

The eligibility criteria section of a clinical trial protocol aims to define the characteristics of the population that can participate in it. Eligibility criteria are important because they can directly affect the results and conclusions of a clinical study. The section is usually stored as a textual *XML *field, and thus the writer is free as far as its format is concerned.

Tables 1, 2 and 3 show examples of the three most common formats of the eligibility criteria section. In Table 1, eligibility criteria are divided in two categories, inclusion and exclusion. Inclusion criteria describe medical conditions or other properties of patients eligible to participate in a clinical trial. In contrast, exclusion criteria are about characteristics of patients that are not allowed to take part. In Table 2, criteria are listed separately for each of a number of aspects concerning eligible patients. In both the formats of Tables 1 and 2, criteria are often multiply indented. On the contrary, in Table 3, criteria are specified in free text paragraphs. Inclusion and exclusion distinctions are verbally stated and criteria are usually described more elaborately and verbosely.

**Table 1:** Sample of eligibility criteria section formatted as a list of inclusion and exclusion criteria

Inclusion Criteria:
- Diabetes mellitus, Type 2
- 25 < BMI < 45 kg/m^2^
- 7,5% < HbA1c < 9%
- Treated with a basal insulin, and at least 1 g metformin daily, for more than 3 months
Exclusion Criteria:
- Type 1 diabetes mellitus
- Treatment with OADs only
- Treatment with thiazolidinediones
- Pregnancy
- Likelihood of requiring treatment during the study period with drugs not permitted by the clinical study protocol
The above information is not intended to contain all considerations relevant to a patient's potential participation in a clinical trial.

**Table 2:** Sample of eligibility criteria section formatted as a list of various aspects

DISEASE CHARACTERISTICS:
- Histologically confirmed adenocarcinoma, including:
- Breast cancer (female), meeting the following criteria:
- Stage I-III disease
- Has undergone complete surgical removal of invasive cancer by mastectomy or lumpectomy
- Newly diagnosed disease
- Scheduled to receive chemotherapy
PATIENT CHARACTERISTICS:
- Life expectancy > 6 months
- Fluent in English
- Not living in a nursing home
- No severe dementia

**Table 3:** Sample of eligibility criteria section in free text format

Patients with COPD: irreversible air-flow limitation (postbronchodilator FEV1/FVC < 70% according to GOLD guidelines). Patients already receiving inhalative therapy can continue their medication. Patients showing a partial reversibility after bronchodilation (postbronchodilator FEV1 increase > 150 ml but < 200 ml) and complaining respiratory symptoms (e.g. dyspnea at exertion) will be treated preoperatively with a short-acting beta-agonist to achieve optimal perioperative conditions.
Patients have to be in clinical stable condition (no symptoms of respiratory tract infection for at least 2 weeks prior to the study).
Patients without COPD: postbronchodilator FEV1/FVC > 70%.

Extracting single eligibility criteria is a prerequisite both for using them as a search filter and for recommending the criteria that best represent a set of documents (Figure 6). Although extraction is straightforward from lists of criteria, such as in formats of Tables 1 and 2, it is very difficult in free text. Apart from deep linguistic processing and use of structured knowledge this task would potentially require a rule-based approach, which would in turn require manual work and would be domain dependent. In addition, scoring the extracted criteria requires that they occur with significant frequency. Without heavy abstraction of surface forms this is highly unlikely to happen.

Deciding whether a criterion refers to inclusion or exclusion is easy only in lists in the format of Tables 1 and 2. Criteria in the format of Table 1 are listed under either inclusion or exclusion headers while criteria in the format of Table 2 should all be considered as inclusive. In format of Table 3, inclusion or exclusion can be expressed in a multitude of ways, impeding recognition.

To overcome these problems, for the task of generating features to narrow down search we chose to extract ontology concepts occurring in the eligibility criteria sections of protocols rather than identify single criteria. We hypothesise that concepts can serve users better than longer full sentences. Biomedical concepts can be identified from free text by ready-made tools. We employed the *UIMA *annotator of *MetaMap *[9], which is a configurable program to discover UMLS Metathesaurus concepts referred to in text. For the recommendation system, we preferred to use full text single criteria occurring in the format of Table 1. Extraction is performed automatically by specialised *UIMA *annotators, that take advantage of bullets, numbering and indentation.

### Recommendation system

The recommendation system aims to suggest eligibility criteria representative of a set of clinical trial protocols. Since the effort required to decide upon the correct criteria for a new trial is significant, especially for inexperienced clinicians, we hypothesize that it is easier to select a set of similar existing trials via the search engine. These documents are then given as input to a recommendation system which in turn outputs a set of ranked eligibility criteria candidates. In essence, the recommendation system scores each eligibility criterion of the input protocols according its importance for the set of chosen documents.

The most important problem in judging the importance of eligibility criteria statistically is sparsity. Since eligibility criteria are in the form of free text, it is extremely rare for an eligibility criterion, i.e. a sentence or a bullet-point, to occur more than once in a set of documents. Decreasing the length of lexical units, from sentences to phrases or even tokens, can be a solution to the sparsity problem. However, such a decision creates other problems. Not all tokens and phrases are important for the task for scoring eligibility criteria and, even worse, unimportant functional words and phrases are more frequent than meaningful ones for the medical domain. To alleviate this, we score the sentences of the eligibility criteria based on the UMLS concepts that they contain. UMLS concepts of eligibility criteria section sentences have been already identified using *MetaMap *[9].

Thus, the eligibility criteria recommendation system scores each sentence in the eligibility sections of the documents selected by the user based on the UMLS concepts that it contains. Given that the user has selected documents of interest, the more frequently a UMLS concept occurs in their eligibility criteria, the most important it is for the document collection and the highest weight it should be assigned. In turn, the more high-weighted concepts a sentence contains, the higher it should be placed in the list of recommendations. However, it is undesirable to favour long sentences, even if they are more likely to contain more concepts. Merging these two requirements into one, each sentence or bullet-point in the eligibility sections of the documents selected by the user is scored by the average of the frequencies of the UMLS concepts that it contains. Concept frequencies are computed within the eligibility sections of the user-selected documents. Eligibility criteria duplicates are removed from the list.

This simple, raw frequency-based statistical computation has been chosen for the eligibility criteria recommendation system, since it is very crucial that it runs quickly for large document collections and parallel requests on a server. The current computation replaced a much more sophisticated and computationally intense mechanism, that was presented in detail in the conference version of this work [10]. The previous mechanism was representing eligibility sentences and bullet-points as mixtures of latent topics and was based on the intuition that a representative set of criteria is associated with topics that are dominant in the input documents. In contrast to the current method, that approach used no ontological resources but instead very intense offline training pre-processing. In addition, the update procedure was rather demanding also the results were of lower quality than the current method. Interestingly, the method in [10] was favouring long criteria segments. The reason lied in the criteria scoring function, i.e. the probability-weighted sum of the topic proportions inferred from the input criteria.

### Indexing

The purpose of this component is to transform each XML-formatted clinical trial protocol into an *Apache Solr *index file. This file should contain all details about the document, since the original protocol is not available during the online function. For example, the content of the protocol with identification *NCT00698698 *that is displayed by *PASC *(Figure 2) is stored in an index file. To extract information that corresponds to the values of pre-selected XML fields an XML parser was employed. The resulting *Apache Solr *index files were indexed by *Apache Solr*, which is a Java-based, highly scalable, open-source search engine platform that supports full-text and faceted search.

### Clusters and cluster labels

This component aims to cluster the search results at each stage of the search process. Since clusters serve as a means of narrowing down search, labelling them is a strong prerequisite. Labels should ideally be short, meaningful and accurate descriptions of the contents of the corresponding clusters. The quality of labels is crucial, given that users may disregard a cluster with a very long or meaningless label even if its content are coherent. Further, speed is also very important because the component is part of the online process. For this purpose, two different algorithms are employed, interchangeably: *Carrot*^2 ^and *UTC*, a new clustering and labelling algorithm based on unigrams and terms.

*Carrot*^2 ^*(search.carrot2.org) *is an open-source Java-based clustering engine that organises search results into thematic categories. It comes together with a component that fetches results from *Apache Solr*. The heart of *Carrot*^2 ^is a soft-clustering algorithm called *Lingo *[11]. *Lingo *uses the *vector space model (VSM) *and *latent semantic indexing (LSI)*. Initially, *VSM *represents each document as a vector whose dimensions are the words that occur in it and the corresponding values are frequencies of occurrence. In succession, *LSI *reduces the dimensionality of these vectors by approximating the original word-document matrix with a limited number of orthogonal factors. Each of these factors represents an abstract concept that occurs in a subset of the documents, but unfortunately cannot directly serve as cluster label, because it does not correspond to a known verbal meaning. For this reason, *Lingo *uses frequent words or sequences as cluster candidate labels. It treats a set of candidate labels as small sized documents using the same *VSM *and projects them to the obtained orthogonal factors. For each abstract concept, the projected values of these candidate labels are used as confidence scores. The phrase with the highest score is assigned to each abstract concept as its cluster label. Finally, documents are assigned to clusters employing standard *VSM*.

Experimentation has shown that *Lingo *sometimes produces long or meaningless cluster labels. The reason probably is that *Lingo *treats terms and documents as equal vectors, thus, long terms are favoured as they are more similar to documents than shorter ones. To address this problem, a new algorithm, *UTC (Unigram and Term-based Clustering)*, was developed based on unigrams and multiword terms. *UTC *projects both terms and documents in a common semantic space instead of using the same *VSM*, thus, is independent of the length of candidate terms and able to produce more meaningful cluster labels. Term extraction algorithms such as *C-Value *have been shown to outperform the raw frequency baseline on biomedical data [7], and thus using terms instead of frequent sequences is expected to increase the quality of cluster labels. *UTC *takes as input terms extracted using the *C-Value *algorithm (subsection "TERMINE: term extraction"). Viewing terms as very short documents allows to represent documents and terms into the same semantic space using *VSM. Tf-idf *weights are computed to assess the importance of terms to documents. Then, *CFRM (Collective Factorization on Related Matrices) *[12,13] is employed to project both terms and documents into a common semantic space. In succession, terms and documents are concurrently clustered by implementing the *k-means *algorithm in the common space. Each term is scored according to its total cosine similarity to the documents occurring in its cluster. Each cluster is labelled by its highest scoring term. In contrast to *Lingo*, *UTC *is a hard clustering algorithm. *UTC *is an application of a spectral method for unsupervised dimensionality reduction, discussed in detail in [14] among others.

To assess *UTC*'s performance versus *Lingo *we conducted experiments. We used as input 1800 protocols that were returned by 9 frequently occurring query words: *asthma*, *breast cancer*, *lung cancer*, *prostate cancer*, *cardiovascular*, *HIV*, *leukemia*, *depression*, *schizophrenia*. The feature space consisted of 14, 000 words and 9, 275 multiword terms. Using 5-fold cross validation, the results were found to be 0.88 separable by nearest neighbour analysis, 0.67 by Fisher linear discriminant analysis, and 0.98 by a linear support vector machine. According to a variety of cluster quality metrics, *UTC *performed better than *Lingo *(Table 4). Moreover, the labels of *UTC *are shorter and easier to understand (Table 5).

**Table 4:** Cluster quality metrics for Lingo and UTC

	*Lingo*	*UTC*
Cluster purity	0.423	0.825
Pairwise cluster contamination	0.644	0.242
Within-cluster similarity	0.363	0.531

The table shows the scores of cluster purity, pairwise cluster contamination and within-cluster similarity achieved by the clustering and cluster labeling algorithms *Lingo *and *UTC*. Experiments used 5-fold cross validation and were performed on 1800 clinical trial protocols containing 9 frequently occurring query words.

**Table 5:** Cluster labels and sizes of the ten larger clusters for Lingo and UTC

*Lingo*	*UTC*
Early stage breast cancer patient (1549)	Lung cancer (661)
HIV infected TB patient (1478)	Depressive symptom (214)
Recurrent major depressive disorder (877)	HIV infection (155)
Androgen independent prostate cancer (817)	Prostate cancer (136)
Schizophrenia/schizoaffective disorder (642)	Breast cancer (127)
Unrelated allogeneic stem cell transplantation (559)	Asthma symptom (111)
Moderate persistent allergic asthma (424)	Cell lung (111)
Others (17)	Antipsychotic medication (106)
Low income innercity (1)	Blood pressure (98)

The table shows labels and sizes of clusters returned by *Lingo *and *UTC *during the experiments performed. They only represent the output of one of the experiments and are included for demonstration purposes.

### Related work

Lately, there has been increased interest in processing clinical trial information. Several systems attempted to transform clinical trial information in computable forms. *CTeXplorer *[15] is a tool that visualises information of randomized clinical trials (*RCTs*) of heterogeneous design. The authors build on the idea that organised representation simplifies and accelerates reviewing and designing trials. Towards the same target, [16] and [17] attempt to extract clinical trial information from free text (e.g. journal articles). The system of [16] employs a text classifier with a weak regular expression matcher while *ExaCT *[17] consists of an information extraction component and a user interface that allows users to assess and modify systems selections.

Significant efforts have been devoted to classifying clinical trials or their parts. [18] utilises shallow semantic parsing to annotate abstracts of *RCTs *meaningful tags. A supervised domain-adaptation approach is adopted. [19] exploits a similar idea in a finer-grained level of syntactic units. The authors classify sentences of *RCT *abstracts in meaningful categories, i.e. introduction, objective, method, result and conclusion, combining text classification and Hidden Markov Modelling techniques. [20] uses *Conditional Random Fields *to select sentences of abstracts that discuss issues of high importance: intervention, participants and outcome measures.

A few systems for writing new clinical trials have been proposed. For example, *WITH *[21] is a tool based on *XML *and on a *relational database management system (RDBMS)*. However, these systems refrain from using text mining and machine learning, in opposition to this work.

Apart from research concerning clinical trials in general, there are several works referring specifically to eligibility criteria. The majority of approaches attempt to represent eligibility criteria formally, so that they are computer-interpretable for further processing. This task is claimed to be crucial both for selecting appropriate candidates for a trial and for identifying trials for similar patient populations. [22] present a review on this issue and identify five major aspects:

- the intended use of computable eligibility criteria

- the classification of eligibility criteria

- the expression language for representing eligibility rules

- encoding eligibility concepts

- modelling patient data

They claim that representation requirements vary for different uses and discuss the implications of the above aspects towards standardization approaches. [23] attempt to formalise eligibility criteria. Instead of using a formal expression language they adopt a less labour-intensive format called *ERGO *annotation. [24] address the problem of determining suitable candidates to participate in clinical trials. The system inputs a set of clinical eligibility criteria in the form of first order predicate logic and locates candidates via their electronic medical records. *ASCOT *analyses eligibility criteria for different purposes; to use them as a means of narrowing down search and for recommending which are the most representative of a set of trials.

## Conclusion

In this paper, we have presented *ASCOT*, a customised search application for clinical trials search. *ASCOT *takes advantage of state-of-the-art text mining, clustering and term extraction technologies to induce valuable metadata relevant to clinical trial protocols and uses them to provide the user with powerful tools to narrow down search. In addition, *ASCOT *is able to suggest eligibility criteria with respect to a set of chosen clinical trials so as to help researchers in composing new clinical trials. Firstly, *ASCOT*'s functionality is discussed accompanied with a selection of screenshots of the user interface. In succession, the architecture is presented and each component is analysed, separately.

In the future, we plan to evaluate *ASCOT *thoroughly. Instead of measuring performance by focussing on the internal functionality of the system as most evaluations of text-mining systems in the past, we intend to conduct a user-centred evaluation. It will be concerned more with determining the performance of the system from a user perspective and assessing how well the system actually fulfils the user's requirements [25]. The strengths, weaknesses and potential improvements of *ASCOT *will be estimated with respect to the following dimensions: functionality, reliability, usability, efficiency and maintainability.

## Competing interests

The authors declare that they have no competing interests.

## Authors' contributions

The first author is the sole developer of ASCOT system and led the writing up of the present paper. The second author has developed the *UTC (Unigram and Term-based Clustering) *algorithm, employed by ASCOT and presented in subsection "Clusters and cluster labels". The third author supervised both preceding authors, is the principal investigator of the clinical trials project in the National Centre for Text Mining (NaCTeM) and provided the research directions for ASCOT.
